# Comparison of molecular mutations of G6PD deficiency gene between icteric and nonicteric neonates

**Published:** 2013

**Authors:** Yadollah Zahedpasha, Mousa Ahmadpour Kachouri, Haleh Akhavan Niaki, Roya Farhadi

**Affiliations:** 1 *Non-Communicable Pediatric Diseases Research Center, Babol University of Medical Sciences, Babol, Iran****.***; 2 *Cellular and Molecular Biology Research Center, Babol University of Medical Sciences, Babol, Iran****.***

**Keywords:** Icter, G6PD deficiency, mediterranean mutation

## Abstract

Jaundice is a common disorder in neonates and one of the provable causes of glucose-6-phosphate dehydrogenase (G6PD) deficiency, some mutation types of which may be associated with severe neonatal icter. The present study has been conducted to compare G6PD mutations in incteric and non icteric neonates. This case-control study was implemented in the NICU and Newborn Ward of Amirkola Children Hospital in 2007-2008. Available sampling approach was used and 50 icteric as well as 50 non-icteric newborns, both with G6PD deficiency, were selected as the case and the control group respectively. G6PD deficiency was diagnosed using FST (Fluorescent Spot Test) method. All samples were first evaluated in terms of Mediterranean mutation and the negative cases were then examined for Chatham mutation; all remaining samples were finally tested for Cosenza mutation. G6PD mutations were compared in the two groups and P-value less than 0.05 was considered significant. In icteric group, 76% were male and 24% were female and in non-icteric group, 70% were male and 30% were female. The mean weight of neonates was 3.2 ± 0.4 kg and 2.8 ± 0.8 kg in icteric and non-icteric groups respectively (p<0.05). In non-icteric group, 54% Mediterranean, 18% Chatham, and 28% Cosenza negative were observed and in icteric group, 56% Mediterranean, 32% Chatham, and 12% Cosenza negative were found; the distribution of Mediterranean and Chatham mutations was not significantly different between the two groups (p>0.05), however, the distribution of rare mutations (Cosenza negative) was significantly different between icteric and non-icteric groups with enzyme deficiency (p<0.05). The mean bilirubin level was not statistically different in Mediterranean (18.5±2.9), Chatham (18.8±2.1) and Cosenza negative (20±4.3) mutations (p> 0.05). Newborns with Chatham mutation have been less in need of exchange transfusions (p <0.05) indicating that rare mutations of G6PD gene may less likely lead to neonatal icter.

G6PD (glucose – 6 – phosphate dehydrogenase) deficiency is the most common enzymatic deficiency of red blood cell, affecting app-roximately 400 million people worldwide (1). Lack of this enzyme increases erythrocyte susceptibility to oxidative stress (2). The disorder was first diagnosed in 1956 and many investigationshave been conducted since then (3); the Electro-phoretic forms of which were identified in 1960 (4). G6PD deficiency is highly different in terms of diversity and distribution; almost 7.5% of people in the world are the carriers of one or two G6PD deficiency genes (5). In an investigation in the city of Babol, the prevalence of the disorder has been reported to be 12.5% in males and 4.1% in females (5). 

Glucose-6-phosphatase gene is located on the distal part of the long arm of chromosome X at Xq28 position (2, 5). With 13 exons and 12 introns, the gene has a length of approximately 18.5 kb, encoding a 59 kDa polypeptide with 515 amino acids; the products of this gene form a number of homodimers or tetramers (5, 7-9). 

Up until now, more than 400 G6PD protein variants have been reported worldwide (5, 10, 11). Published list of biomedical G6PD variants diagnosed is periodically being updated (4, 9, 12). One of the most common G6PD variants is the Mediterranean type which is often associated with favism (13-15). 

Studies conducted in Iran – in Mazandaran Province and Sari city – by Mesbah et al., showed that the prevalence of Chatham mutation was higher in this region compared with other parts of the world between three different G6PD polymorphic variants, including 66.2% Medi-terranean, 27% Chatham and 6.75% Cosenza (16), and, hence, the prevalence of all G6PD types in this region is more similar to that in Italy in comparison with other Mediterranean countries (16). Regarding the studies performed, Mediterranean G6PD is the most common mutation in Iran as well as other tropical and subtropical regions (5, 13, 15-17). Neonatal icter is the most common clinical manifestation of G6PD deficiency (18). Icter is developed in one third of newborns with the above-mentioned deficiency (1). If not treated timely, neonatal icter can lead to kernicterus, cerebral palsy, and death (3). In another study by Pietra Pertosa et al. in 2001, severe clinical phenotypes have been reported in carriers of Mediterranean mutation and/or A- variants (19). 

Considering G6PD deficiency, especially in the Northern regions, and its association with neonatal icter and related complications, and the fact that no study has been conducted in Iran in terms of the relationship between enzyme-associated specific DNA mutations and clinical manifestations in neonatal age group, the present study aimed to investigate three common G6PD gene mutations in Mazandaran Province in neonatal age group and its association with the incidence and the severity of neonatal icter.

## Materials and Methods

**Subjects**

This case-control study was implemented in the NICU and Newborn Ward of Shafizadeh Children Hospital of Amirkola in 2007-2008. Available sampling approach was used and regarding P=0.6 (the proportion of the most common mutations in G6PD patients) (13) and according to statistical calculations, 50 icteric neonates with G6PD deficiency in need of treatment with phototherapy and exchange transfusion were selected as the case and 50 non-icteric newborns with G6PD deficiency hospitalized in Newborn Ward and/or NICU were selected as the control for determination of molecular mutations. Each patient was provided a questionnaire in which information on weight, age, gender, diagnosis and bilirubin level were measured and the type of treatment used, duration of hospitalization, newborns′ blood group and Rh were recorded.

**Procedure for detection of G6PD mutations Sample Collection**

3mL of peripheral blood was taken from each neonate after parental consent and was collected in EDTA-containing tubes; G6PD deficiency was measured using FST (Fluorescent Spot Test) method and bilirubin was calculated using DSA (Diazo) approach. All samples were first evaluated for Mediterranean mutation and negative cases were then tested for Chatham mutation and the remaining negative cases were examined for Cosen-za mutation.

**DNA extraction**

Alkalin lysis method was used for the extrac-tion of genomic DNA from 500 µl (0.5 ml) of blood.

**DNA amplification**

For the evaluation of each of the above mutations, mutation-related region in G6PD gene was first amplified for all DNA samples using PCR approach.

To determine the Mediterranean mutation, G6PD exon 6 was amplified by polymerase chain reaction using Forward 5'-CCCCGAAGAGGATT CAAGGGGGT-3' and Reverse 5'-GAAGAGTAGC CCTCGAGGGTGACT-3' primers (MWG Co, Germany). PCR reaction was performed in a 100- µl volume; for this purpose, 10µl of buffer, 6µl of 50mM MgCl2, 1µl of 25mM dNTPs, 2µl of 10 pmoles/ µl of each Forward and Reverse primers and 3.5 units of Taq DNA polymerase (Roche Co, Germany) were added to 2µl of patient′s DNA samples. Exon 6 of G6PD gene was amplified with a thermal cycler (Techne/Techgene, England). For this purpose, DNA first underwent one round of denaturation at 94 °C for one minute. It was then subjected to two rounds at 94°C for 1 min, 55°C for 1 min and 30 sec and 68 °C for 1 min. Afterward, DNA underwent 36 rounds respectively at 94, 55, and 68 degree centigrade, each for 30 sec. In the end, DNA was subjected to one round of final extension at 68° C for 5 min. After this step, PCR products obtained were electrophoresed on 1.5% agarose gel; the appearance of a 583-nucleotide band was indicative of correct DNA replication.

For the determination of Chatham mutation, Exon 9 of G6PD gene was amplified by PCR using Chat F and Chat R primers (MWG Co, Germany). PCR reaction was performed in a 50-µl volume. For this purpose, 5 µl of buffer, 3 µl MgCl2 (50 mM), 0.5 µl dNTPs (25 mM), 1 µl of each Chat F 5'- CAAGGAGCCCATTCTCTCCCTT-3' and Chat R 5'-TTCTCCACATAGAGGACGACGG-3' primers, and 0.35 µl of 5 unit Taq (Gene Fanavaran Co.) were added to 0.75 µl of DNA. Exon 9 of G6PD gene was amplified by the thermal cycler (Techne/Techgene, England). For this purpose, DNA was first subjected to one round at 94 °C for 2 min. It then underwent ten rounds at 94°C for 30 sec, 67.5°C for 1 min, and 25 rounds at 94, 64, and 72 degree centigrade respectively, each for 1 min. In the end, DNA was subjected to one round of final extension at 72° C for 5 min. After this step, PCR products obtained were electrophoresed on 1.5% agarose gel; the appearance of a 208-nucleotide band was indicative of correct DNA replication.

To determine Cosenza mutation, genomic DNA was amplified by PCR using Cos F and Cos R primers (Bioneer Co, Korea). PCR reaction was performed in a 50-µl volume. For this purpose, 5µl buffer, 3µl Mgcl2, 0.5µl dNTPs, 1µl of each Cos F 5'-GCAGCCAGTGGGATCAGCAAG-3' and Cos R 5'-GGCAAGGAGGGTGGCCGTGG-3' primers, and 0.3µl of 5-unit Taq (Gene Fanavaran Co.) were added to 1µl of patient′s DNA. Amplification of this region of G6PD gene was performed using the thermal cycler (Techne/Techgene, England). For this purpose, DNA was first subjected to one round at 94 °C for 1 min. It then underwent 38 rounds at 94, 66, and 72 degree centigrade respectively, each for 1 min. In the end, DNA was subjected to one round of final extension at at 72° C for 4 min. After this step, PCR products were electerophoresed on 1.5% agarose gel; the appearance of a 548-nucleotide band was indicative of correct DNA replication.

**Restriction analysis**

**Fig 1:**
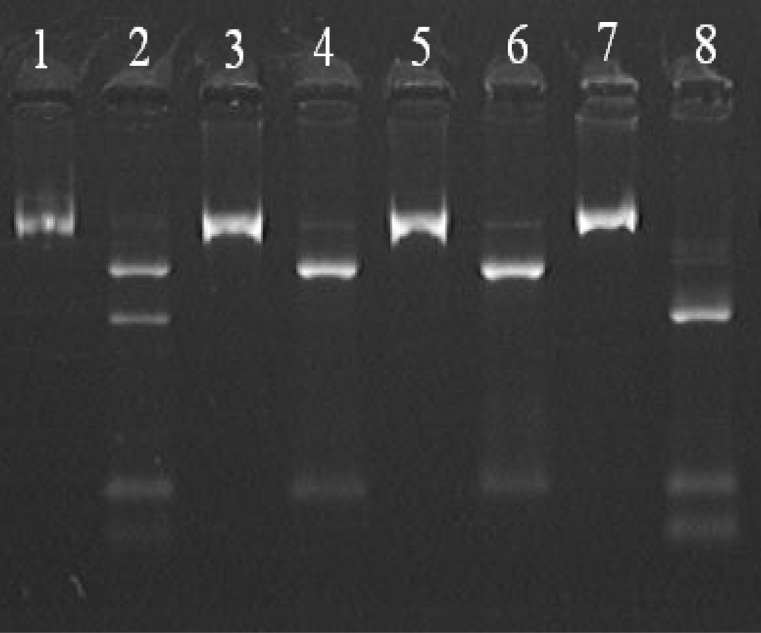
PCR products digested with Mbo II restriction enzyme. Lanes 1, 3, 5, 7 show the PCR products before the effect of restriction enzyme (583-neucleotide band is visible) and Even columns are related to PCR products after the enzymatic diges-tion. Lanes 4, 6 show samples without Mediterranean mutation. Lane 2 shows female heterozygote with Mediterranean muta-tion. Lane 8 shows a male with Mediterranean mutation

For Mediterranean mutations, PCR products were incubated with Mbo II (Fennentas Co., Russia) for 17 hours at 37°C. Digested products were electrophoresed on 2% agarose gel for 2 hours, and the image of each gel was captured in the end. In cases of no Mediterranean mutation and following enzymatic digestion, 60-, 279- and 120-nucleotide bands were visible, and in the presence of mutation, 379-nucleotide band would be cleaved into 276- and 103- nucleotide bands. In heterozy-gous specimens, 60, 120, 276, 103 and 379 samples could be observed (Fig.1).

**Fig 2:**
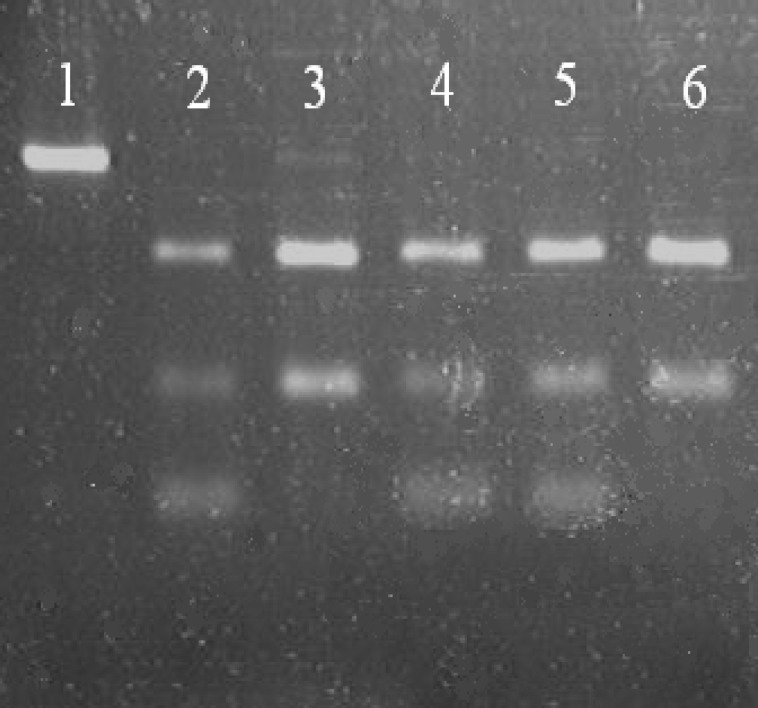
PCR products digested with Bstx1 restriction enzyme. Lane 1 shows the PCR products before the effect of restriction enzyme (208-neucleotide band is visible). Lanes 3, 6, show lack of Chatham mutation. Lanes 2, 4, 5 show samples with Chath-am mutation in carrier females.

For Chatham mutation, PCR products were incubated with Bstx1 restriction enzyme (Fennentas Co, Russia) for 17 hours at 37°C. Digested products were electrophoresed on 2% agarose gel for 2 hours, and the image of each gel was captured afterwards. In cases of no Chatham mutation and after enzymatic digestion, 78-bp and 130-bp bands are visible, and in the presence of mutation, 130-bp band will be cleaved into 100-bp and 30-bp bands (Fig.2). 

For Cosenza mutation, PCR products were incubated with Bsu361 restriction enzyme (Fermentas Co., Russia) at 37°C for 17 hours. Digested products were electrophoresed on 2% agarose gel for 2 hours, and the image of each gel was captured. In cases of no Cosenza mutation and following enzymatic digestion, 548-bp band is visible, and in the presence of mutation, the band will be cleaved to 232-bp and 316-bp bands. 

Data obtained were analyzed by SPSS 15 statistical software using Chi-square and ANOVA, and p<0.05 was considered significant.

## Results

In the present study conducted on 50 icteric and 50 non-icteric neonates with G6PD deficiency, 38 subjects (76%) were male and 12 (24%) were female in the former, and 45 (70%) were male and 15 (30%) were female in the latter group. The mean weight of icteric and non-icteric neonates was 3.2 ± 0.4 kg and 2.8 ± 0.8 respectively. Non-icteric neonates were in the age range of 1 to 15 days with the mean of 6.8 ± 4.5 days. Eleven patients (22%) in the icteric group had a positive family history. The mean hemoglobin level was 16.1 ± 2.3 mg/dl in icteric group. The age of onset of icter was 2.7 ± 1.2 days, ranging from 6 to 8 days, and the mean bilirubin level was 18.3 ± 2.8 mg/dl in this group. The type of treatment used for icteric patients was phototherapy in 30 neonates (68%) and exchange transfusions in 16 cases (72%). The mean hospital stay was 4.4 ± 1.2 days, ranging from 2 to 8 days. Mediterranean mutation was the most frequent mutation observed in icteric and non-icteric groups, while Cosenza mutation was found in none of the samples; the distribution of rare mutations of G6PD gene was significantly higher in non-icteric compared to icteric neonates with G6PD deficiency (Table 1).

In total, out of 55 newborns with Medi-terranean mutation, 42 subjects (76.4%) were male and 13 (23.6%) were female; out of 3 neonates with Chatham mutation, 15 cases (16%) were male and 10 (40%) were female (P=0.091), and out of 20 patients with Cosenza negative, 16 (80%) were male and 40 (20%) were female. The mean bilirubin levels and hospital stay showed no significant difference in icteric group for different types of mutation (Table 2).

The distribution of treatment type in icteric newborns for different types of G6PD gene mutations revealed no significant difference between Cosenza negative and Mediterranean mutation; however, it was statistically different in terms of Chatham mutation, and newborns with this mutation have been less in need of exchange transfusion (Table 3).

**Table 1:** Frequency of G6PD gene mutations in icteric and non-icteric neonates with G6PD deficiency

**Group Mutation**	**Non-icteric** **Frequency (%)**	**Icteric** **Frequency (%)**	**P-Value**
Mediterranean	27 (54%)	28 (56%)	0.841
Chatham	09 (18%)	16 (32%)	0.106
Cosenza negative *	14 (28%)	06 (12%)	0.046
Total	50 (100%)	50 (100%)	

* Those cases evaluated in terms of Cosenza mutation and all were negative are probably related to other G6PD gene mutations

**Table 2:** The mean and standard deviation of bilirubin level and the duration of hospitalization in icteric neonates for different types of mutation

**Parameter**	**Mutation**	**Number**	**Mean ± SD**	**P-Value**
Bilirubin (mg/dl)	Mediterranean	28	18.5 ± 2.9	0.518
	Chatham	16	18.8 ± 2.1	
	Cosenza negative	06	20 ± 4.3	
Hospitalization duration (day)	Mediterranean	28	4.7 ± 1.2	0.066
	Chatham	16	3.8 ± 1.1	
	Cosenza negative	06	4.8 ± 1.9	

**Table 3:** Frequency of treatment types in icteric neonates for different types of G6PD gene mutations

**Mutation**	**Phototherapy** **Frequency (%)**	**Exchange transfusion** **Frequency (%)**	**Total**	**P-Value**
Mediterranean	18 (64.3%)	10 (35.7%)	28 (100%)	0.131
Chatham	12 (75%)	4 (25%)	16 (100%)	0.046
Cosenza negative	4 (66.6%)	2 (33.3%)	6 (100%)	‒

## Discussion

The results of the present study demonstrated that Mediterranean mutation was the most frequent mutation in icteric and non-icteric neonates with G6PD deficiency; this mutation has been observed in more than half of the newborns in both groups, between which the relative distribution was not statistically different; although the distribution of Chatham mutation was higher in icteric neonates, the difference was not significant (Table 1). Likewise, in an investigation in Italy on 23 infants with severe icter and G6PD deficiency, 39 cases of Mediterranean mutation have been reported (20). Comparison between the present study and other investigations indicate that the mutations observed in icteric newborns with G6PD deficiency have not been significantly different with those in non-icteric neonates with the same deficiency, and Mediterranean type of mutation has similarly been the most common mutation in non-icteric population with mentioned deficiency. Higher frequency of Cosenza negative in non-icteric compared to icteric group is the other notable point in the present study, indicating that the frequency of rare G6PD mutations is lower in icteric compared with non-icteric group (Table 1). What distingui-shes the present research is addressing neonatal jaundice among icteric and non-icteric newborns for the first time in Iran since the relationship between the mutation of G6PD gene and clinical manifestation have not been discussed so far in this country. In this study, the mean serum bilirubin level and hospital stay in icteric patients was not statistically significant for different types of mutation (Table 2). Similar to the present study, in an investigation by Ainoon et al. in 2004 on 128 Chinese male newborns with G6PD deficiency, no difference has been found between the incidence of neonatal icter, the mean serum bilirubin level and the percent of newborns in need of phototherapy and duration of phototherapy between the two most common types of G6PD gene mutations (21). Moreover, in another study by Pietra Pertosa et al. in 2001, more severe clinical phenotype has been reported in the carriers of Mediterranean mutation or A- variants (19). In the present study, the percent of exchange transfusion was higher in Mediterranean mutation compared with other mutations; however, the difference was not statistically significant. In addition, in this study neonates with Chatham mutation have been less in need of exchange transfusion and their clinical phenotype has been milder (Table 3). Studies conducted in Sari have revealed that the prevalence of Chatham mutation is higher in this region of Iran compared with other parts of the world and, hence, the prevalence of G6PD variants in this area is similar to that in Italy compared with other Mediterranean countries (16). Regarding the researches implemented, Mediterranean G6PD is the most common mutation in Iran and other tropical and semi-tropical regions (17). 

Considering cases of Cosenza negative in our study, further evaluation on other mutations of G6PD gene and studies with larger sample size are recommended to determine the severity of icter in Mediterranean and the incidence of Chatham mutations. 
